# Seasonal Changes in Circadian Peripheral Plasma Concentrations of Melatonin, Serotonin, Dopamine and Cortisol in Aged Horses with Cushing’s Disease under Natural Photoperiod

**DOI:** 10.1111/j.1365-2826.2008.01751.x

**Published:** 2008-08

**Authors:** S J A Haritou, R Zylstra, C Ralli, S Turner, D J Tortonese

**Affiliations:** * Pegasus Equine Diagnostics Ltd, BioCity NottinghamNottingham, UK; † Chine House Veterinary GroupLoughborough, UK; ‡ Department of Anatomy, University of BristolBristol, UK

**Keywords:** Cushing’s, laminitis, serotonin, melatonin, dopamine, cortisol

## Abstract

Equine pituitary pars intermedia dysfunction (PPID) is a common and serious condition that gives rise to Cushing’s disease. In the older horse, it results in hyperadrenocorticism and disrupted energy metabolism, the severity of which varies with the time of year. To gain insight into the mechanism of its pathogenesis, 24-h profiles for peripheral plasma melatonin, serotonin, dopamine and cortisol concentrations were determined at the winter and summer solstices, and the autumn and spring equinoxes in six horses diagnosed with Cushing’s disease and six matched controls. The nocturnal rises in plasma melatonin concentrations, although different across seasons, were broadly of the same duration and similar amplitude in both groups of animals (P > 0.05). The plasma concentrations of cortisol did not show seasonal variation and were different in diseased horses only in the summer when they were higher across the entire 24-h period (P < 0.05). Serotonin concentrations were not significantly affected by time of year but tended to be lower in Cushingoid horses (P = 0.07). By contrast, dopamine output showed seasonal variation and was significantly lower in the Cushing’s group in the summer and autumn (P < 0.05). The finding that the profiles of circulating melatonin are similar in Cushingoid and control horses reveals that the inability to read time of year by animals suffering from Cushing’s syndrome is an unlikely reason for the disease. In addition, the results provide evidence that alterations in the dopaminergic and serotoninergic systems may participate in the pathogenesis of PPID.

Several variants of equine Cushing’s disease have been described (1), with pituitary pars intermedia dysfunction (PPID) (2) being the most widely recognised. Cushing’s disease is often characterised by hypertrophy and hyperplasia of the pituitary pars intermedia, and is thought to result from a reduction in dopamine synthesis or the degeneration of the periventricular hypophyseal dopaminergic neurones (3). The clinical signs include hirsutism, polydipsia, polyuria, increased protein catabolism (decreased muscle mass), glucose intolerance and insulin refractoriness, suppression of the immune system and general lethargy (1). The disease is progressive and occurs mainly in aged horses and ponies. Many afflicted animals develop laminitis, a systemic disease (4, 5) with a seasonal onset, being most often observed in the autumn.

Currently, the aetiology, pathogenesis and treatment of PPID and Cushing’s syndrome are a matter of intense debate and there is no reliable prophylaxis, or remedy, for the disease (2). Treatments are generally given to ameliorate the clinical signs and may include dopamine agonists, serotonin (5-hydroxytryptamine/5-HT) antagonists (6, 7) and/or nonsteroidal anti-inflammatory drugs and blood vessel dilators where laminitis has developed (4). The administration of dopamine and its agonists was reported to decrease plasma concentrations of pro-opiomelanocortin (POMC)-derived peptides (8). It has also been demonstrated that vasoactive amines with molecular structures similar to those of serotonin cause digital vasoconstriction and release serotonin from equine platelets *in vitro* (9), and that 5-HT_2_ receptors mediate the effects of stress on the activity of dopaminergic neurones in mice (10). Moreover, administration of an inhibitor of 3-hydroxysteroid dehydrogenase, which reduces cortisol production, appeared to be successful in decreasing the incidence of laminitis in horses (11). Finally, in many seasonally breeding mammalian species living in temperate zones, photoperiod is the major environmental cue that controls annual physiological cycles via both the effects of melatonin upon the hypothalamic-pituitary axis (12) and dopamine in an inhibitory role (13). Hence, all four neurohormones may be involved in the development of PPID as a seasonally variable and known dopamine-deficient condition.

It has been observed that early diagnosis of Cushing’s disease may be obscured by the overlap between phenotypic changes, as well as by age and accompanying diseases (14). Because PPID is most prevalent in older horses, it is important to consider the possibility that concurrent phenotypic changes occur as a result of gerontological alterations in neurohormonal concentrations. These latter involve a progressive loss of central and peripheral neurotransmitter and hormone sensitivity over time (15, 16), as a result of degeneration of the pineal gland, and could result in decreased serotonin and dopamine concentrations in peripheral blood.

The present study aimed: (i) to test the hypothesis that the aged horse cannot ‘read’ the time of the year because of deficiency in melatonin production (and thus its circulating plasma concentration), resulting in an impaired 24-h profile, and (ii) to examine the link between the presence or absence of the clinical signs of Cushing’s syndrome and differential peripheral plasma concentrations of serotonin, dopamine and cortisol across the year in aged horses and ponies.

## Materials and methods

### Animals

The chosen population represented a random selection of breeds or types of horses and ponies from varied geographical locations throughout the UK. The animals selected for the study remained at the same site throughout the observation period; they were aged 21–36 years, had been retired from work, and were kept at grass. During the December and March sampling sessions, they were brought into stables from 16.00 h to 07.30 h. This regimen was started a minimum of 4 weeks in advance of the session as part of their regular winter management. The study was conducted without making any changes to the animals’ normal feeding and management regimen. All experimental protocols were carried out in strict accordance with UK Home Office regulations.

### Experimental strategy and design

Two groups were constructed from the population: a ‘diseased’ (or Cushingoid) and a ‘control’ group. The former consisted of horses and ponies with a veterinary history that strongly implied the presence of Cushing’s disease, or where external examination confirmed the same. The control group was selected from the same population with each member matching an individual in the disease group as closely as possible in breed/type, gender, weight and height. Static hormonal and biochemical determinations from a blood plasma or serum sample obtained via jugular venepuncture were performed to confirm the assignment. The predominance of the following values indicated correct allocation to the Cushing’s group: adrenocorticotrophic hormone > 50 pg/ml, cortisol > 155 nmol/l, glucose > 7.50 mmol/l, triglycerides > 0.76 mmol/l, cholesterol > 2.84 mmol/l, magnesium < 0.71 mmol/l and alkaline phosphatase > 326 U/l (5, 17, 18). From the starting pool of animals, six matched pairs with a good expectation of survival to the end of the study and negligible anxiety during jugular venepuncture were selected.

Blood samples were collected at 27-h intervals over 4 × 10-day periods, each scheduled to span consecutive solstices and equinoxes throughout a year. Day 1 commenced at 13.00 h and was a control for day 9, also at 13.00 h. All sampling during hours of darkness was conducted using red light to minimise any effects on the pineal gland. Different operators were used to draw samples from different horses across both groups to reduce bias. Because the horses and ponies were at grass, they were caught and brought into catch-up areas in the hour preceding the sampling start time; each individual was secured and given a minimum of 20 min in a calm state before sampling commenced. The samples were then collected from the jugular vein via needle and syringe, gently transferred to Vacutainer: Becton, Dickson and Company, Franklin Lakes, NJ, USA spray-coated K2EDTA plastic tubes (without using a vacuum, i.e. not through the cap) and kept within insulated boxes at 2–8 °C. They were promptly centrifuged at 1300 ***g*** for 12 min within 2 h of collection, immediately processed and stored short-term between −25 and −30 °C. Platelet-poor plasma samples for serotonin determinations were centrifuged at 4500 ***g*** for 10 min. Long-term storage in the laboratory was at −75 °C.

To be able to examine individual circadian profiles in accordance with the UK Animal Welfare regulations, the experimental design included a consistent time slot for each animal on each sampling day. For example, Horse 1 had a sample drawn at 13.00 h on day 1. This individual would then have a sample drawn at 16.00 h on day 2. Similarly, Horse 2 had a sample drawn at 13.06 h on day 1 and at 16.06 h on day 2. Thus, the time slots were 6 min apart and were maintained for each individual over the course of the study. Using this design, the results from all horses on day 1 were designated as ‘time point 13.00 h’, from day 2 as 16.00 h, from day 3 as 19.00 h, and so on, until day 9 when blood collection at 13.00 h (control for day 1) terminated the experiment. Because the natural rhythms of the horses were being observed in the context of daylight-saving time (the UK yearly shifts between GMT and BST and horses adapt to management and feeding changes associated with this), all sampling times are as would be expected in the UK (i.e. GMT for December and March, BST for June and September).

### EDTA plasma radioimmunoassays (RIA)

Commercial ^125^I RIA kits were obtained for quantitative determinations of melatonin, serotonin and dopamine (Labor Diagnostika Nord GmbH & Co, KG, Nordhorn, Germany). These kits were validated for use with equine plasma via standard parallel and serial dilution techniques, and also by running test samples at the same concentrations within the same assay kit and across different assay kits of the same product code. Analytical sensitivity, as reported by the manufacturer, was 0.4 pg/ml (400 μl version), 10 ng/ml and 6 pg per sample volume unit extracted (ml) for the melatonin, serotonin and dopamine kits, respectively.

Endogenous melatonin was removed by adsorption to activated charcoal and an ‘Equalising Reagent’ (i.e. melatonin-free biological liquid), in this case equine plasma, was then used to equalise the assay matrix of standards and untreated samples. All samples, including the standards, were digested by a protease to reduce nonspecific binding. To prepare samples for serotonin determination, an acylation reagent was used to convert the serotonin into *N*-acylserotonin quantitatively. Dopamine was extracted using a cis-diol specific affinity gel, acylated to *N*-acyldopamine and then converted enzymatically during the detection procedure into *N*-acyl-3-methoxytyramine.

Validation of the melatonin kit was performed with the volume used for hormone quantification in the samples from the study (400 μl). Intra- and inter-assay coefficients of variation (CV) were in the range 4.2–19.7% and 9.4–22%, respectively, and the mean recovery was 100.2%. Mean linearity was 84.9%. Testing of the dopamine kit was carried out to confirm its reliability in measuring the low content of dopamine in equine plasma. The intra-assay CV was in the range 10.5–12.4% and the inter-assay CV was in the range 10.1–17.8%. The analytical recovery of dopamine was estimated at eight different concentrations by using the theoretically expected and the actually measured values; the mean recovery was 92.3%. Linearity was determined using seven different dilutions of a serum sample with serum equalising reagent; this gave a mean linearity value of 98.9%.

Cortisol was quantified via an enzyme-linked immunosorbent assay using commercial kits from DRG Instruments GmbH (Marburg, Germany) that were validated for use with equine plasma in a manner similar to that for the RIA kits. Intra- and inter-assay CVs were in the range 3.2–8.1% and 6.5–7.7%, respectively. The mean recovery was 97.1% and mean linearity, 98.1%.

Prior to assay, the samples were thawed at room temperature (approximately 20 °C), immediately centrifuged at 6 °C and 3000 ***g*** for 5 min to remove any particulates, and the supernates assayed without delay. The assay kit protocols, timescales and quality control measures were strictly adhered to. As far as possible, all samples from the same horse or pony were analysed at the same time in the same kit for each sampling period.

### Statistical analysis

All analyses were carried out by QI Statistics Ltd (Reading, UK) using SAS software (Statistical Analysis System, Cary, NC, USA). In the first instance, the control days (i.e. days 1 and 9) were compared using the difference in adjusted mean scores between Time 1 (13.00 h on day 1) and Time 9 (13.00 h on day 9) for each condition (control or Cushingoid) group via a t-test. Overall, there was no evidence for a significant difference between the average hormone concentrations on either of the two control days for any of the seasonal sampling periods. Thus, it was assumed that the result on each sequential day was reliable.

Data were shown to be normally distributed and were statistically analysed in five stages. Stage 1: Due to the involvement of multiple variables in this study, a multifactorial (three-way) split-plot anova was carried out initially to examine the effects of, and interaction between, condition (normal versus Cushingoid), season and time of day for each hormone; condition, season and the interaction between condition and season were applied to the main plot, whereas time, and interaction between time and condition and time and season were applied to the sub-plot. The hormonal ratios serotonin : melatonin and dopamine : melatonin were also analysed in this manner (the ratio directions were selected to minimise the effect of zero or missing data in denominators). Stage 2: The aforementioned original analysis of each hormone was followed by individual split-plot (two-way) anovas with repeated measurements over time for each season; in this case, condition was applied to the main plot, whereas time and the interaction between time and condition were applied to the sub-plot. Stage 3: Due to the diurnal variations in magnitude of some hormones, analyses of the light and dark periods were then carried out separately; the photophase and scotophase were defined using the sunrise and sunset times for the latitude and longitude of the site. Stage 4: Finally pairwise comparisons of the differences in adjusted means at each time point were conducted for each hormone in each season using a t-test. Stage 5: In addition to the above analyses, the diurnal rhythms in hormone concentrations and the hormonal ratios previously specified were examined by cosinor analysis. The cosinor model was fitted using the proc nlin (nonlinear procedure) of SAS to take into account the amplitude, the extent of the cyclic variation around the average value and the acrophase. Subsequently, anova was carried out on the shape parameters using the proc gln (general linear model procedures) of SAS. All graphical data are presented as adjusted means ± SEM.

## Results

**Fig. 1:**
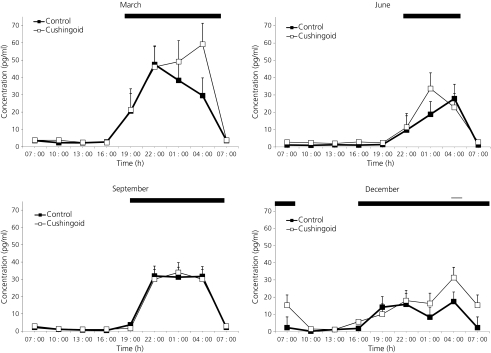
Twenty-four-hour patterns of blood plasma concentration of melatonin in aged horses with Cushing’s disease (light line) and aged control animals (dark line) at four times of the year. Values at each time point represent the mean ± SEM. Sunset to sunrise is indicated by the solid bar above the graphs. The 24-h sample collections began at 13.00 h, but for clarity results are plotted starting at 07.00 h.

The 24-h profiles of melatonin for control and Cushingoid horses in each season are shown in Fig. 1. A highly significant effect of time of year (month) was observed in the total daily secretion of melatonin (P < 0.002); similarly, the 24-h profile was affected by month (P < 0.02). By contrast, no differences were detected between control and diseased horses for either the total melatonin output or its 24-h profile in any month (anova: P > 0.05). This was corroborated by cosinor analysis, which showed no significant differences between the two groups of animals for either the acrophase or the amplitude of the nocturnal peak (P > 0.05).

**Fig. 2:**
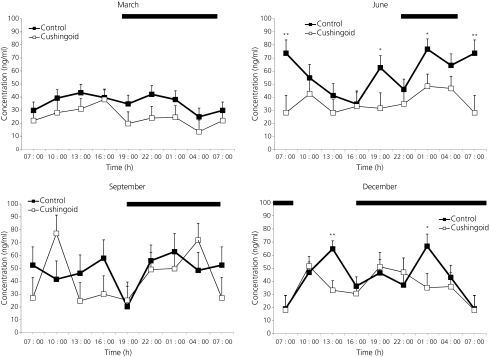
Twenty-four-hour patterns of blood plasma concentration of serotonin in aged horses with Cushing’s disease (light line) and aged control animals (dark line) at four times of the year. Values at each time point represent the mean ± SEM. Sunset to sunrise is indicated by the solid bar above the graphs. The 24-h sample collections began at 13.00 h but, for clarity, the results are plotted starting at 07.00 h. Pairwise comparisons of means at specific time points were performed and significant differences are indicated by asterisks; *P < 0.05, **P < 0.01.

The 24-h peripheral plasma concentrations of serotonin are displayed in Fig. 2. The total secretion within a day was not influenced by time of year. However, the 24-h profiles differed across seasons (time × month interaction P < 0.02). Significant daily changes were detected in June (P < 0.05) and December (P < 0.01). A tendency was also noted for the effect of condition (i.e. control versus Cushingoid) on both total serotonin (P = 0.07) and the 24-h profile itself (P = 0.06). Indeed, concentrations of serotonin were significantly lower at certain times during both the light and dark phases of the 24-h light/dark cycle (P < 0.05) in Cushingoid animals during summer (June) and winter (December).

**Fig. 3:**
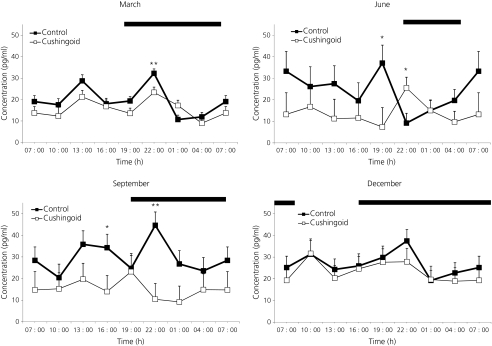
Twenty-four-hour patterns of blood plasma concentration of dopamine in aged horses with Cushing’s disease (light line) and aged control animals (dark line) at four times of the year. Values at each time point represent the mean ± SEM. Sunset to sunrise is indicated by the solid bar above the graphs. The 24-h sample collections began at 13.00 h but, for clarity, the results are plotted starting at 07.00 h. Pairwise comparisons of means at specific time points were performed and significant differences are indicated by asterisks; *P < 0.05, **P < 0.01.

The total amount of dopamine released within the 24-h period was significantly affected by season (P < 0.05) and markedly different between control and Cushingoid horses (P < 0.001). As shown in Fig. 3, diseased animals exhibited significantly lower plasma concentrations at specific times of day in March, June and September. There was a tendency for effect of condition on the overall 24-h profile to be influenced by season (time × condition × month interaction; P = 0.07), with significant time × condition interactions when the profiles were analysed separately for the dark and light phases of the 24-h cycle (P < 0.05). Finally, cosinor modelling detected a tendency (P = 0.08) for a phase shift in the dopamine acrophase in diseased animals with a peak just over 3 h later than in controls.

**Fig. 4:**
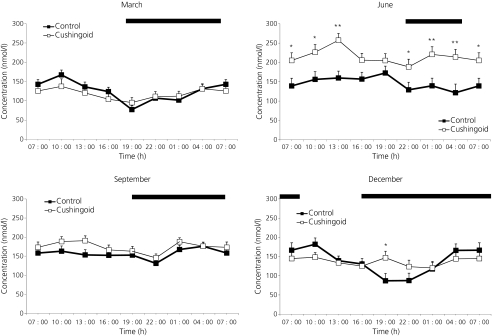
Twenty-four-hour patterns of blood plasma concentration of cortisol in aged horses with Cushing’s disease (light line) and aged control animals (dark line) at four times of the year. Values at each time point represent the mean ± SEM. Sunset to sunrise is indicated by the solid bar above the graphs. The 24-h sample collections began at 13.00 h but, for clarity, the results are plotted starting at 07.00 h. Pairwise comparisons of means at specific time points were performed and significant differences are indicated by asterisks; *P < 0.05, **P < 0.01.

The multifactorial anova showed that overall amounts of cortisol secreted during a day and its 24-h profiles were not affected by season or condition (P > 0.05 for both). Similarly, cosinor analysis revealed no differences in either the acrophase or amplitude (P > 0.05). Because it was felt that the lack of significant differences was due, at least in part, to the interanimal variability, individual anovas for each season were carried out. They revealed that, in June (Fig. 4), plasma hormone concentrations in Cushingoid horses were significantly higher at almost all time points (P < 0.01).

A significant effect of season was detected on the ratios of the total daily secretions of serotonin : melatonin (P < 0.05) and dopamine : melatonin (P < 0.01) and the 24-h profiles for both ratios were significantly affected by month (P < 0.001). Moreover, the ratios of serotonin : melatonin and dopamine : melatonin total secretions were different in Cushingoid and control horses (P < 0.001 and P < 0.005, respectively), as were their profiles in different seasons (condition × season interaction P < 0.001 for both). Values of the two ratios that differed significantly between control and diseased horses at different times of the day in the four seasons of the year are summarised in Table 1. Cosinor modelling showed a significant change in the amplitude for the dopamine : melatonin (P < 0.02) and an almost significant change in serotonin : melatonin (P = 0.059). In both cases, the Cushingoid horses had much lower amplitude values than did the controls.

**Table 1:** Neurohormone Concentration Ratios in Cushingoid and Control Horses Across all Seasons.

		Ser :Mel (ng/ml : pg/ml)		Dop :Mel (pg/ml : pg/ml)	
			
Date	Time (h)	Control	Cushingoid	Control	Cushingoid
March	10.00	27.5 ± 6.69	4.61 ± 9.72*	10.8 ± 2.84	2.22 ± 4.13*
	13.00			16.6 ± 2.81	9.50 ± 3.00*
	19.00	6.73 ± 1.15	2.11 ± 1.68**		
	22.00	6.18 ± 1.42	1.30 ± 1.68**	7.60 ± 1.54	1.17 ± 1.82***
	01.00	7.71 ± 1.42	0.80 ± 1.68***		
June	07.00	72.0 ± 16.1	7.50 ± 18.1***	34.0 ± 12.2	4.99 ± 11.46*
	13.00	50.6 ± 12.6	17.8 ± 13.6*	28.6 ± 9.49	5.27 ± 10.3***
	19.00			37.8 ± 12.2	5.03 ± 10.3**
	22.00	14.6 ± 2.71	8.43 ± 2.86*	2.85 ± 2.03	8.03 ± 1.96*
	01.00	9.49 ± 2.42	1.71 ± 3.36*		
	04.00	14.8 ± 2.71	2.31 ± 2.86***	5.38 ± 2.03	0.84 ± 1.96*
September	16.00	291 ± 65.7	18.7 ± 68.3***	173 ± 41.4	10.2 ± 56.5**
	22.00	9.53 ± 1.84	2.66 ± 1.99**	7.39 ± 1.08	0.50 ± 1.28****
	01.00	8.01 ± 2.12	1.51 ± 1.99**		
December	07.00	11.4 ± 2.21	4.29 ± 2.34**	12.3 ± 2.28	1.73 ± 2.70***
	10.00	414 ± 61.0	39.3 ± 49.1****	323 ± 60.2	17.1 ± 56.8****
	22.00	10.6 ± 2.21	2.45 ± 2.34**	9.97 ± 2.28	4.28 ± 2.70*
	01.00	15.3 ± 1.98	1.77 ± 2.34****		
	04.00	9.40 ± 1.98	2.97 ± 2.34**		

Values are adjusted means ± SEM for statistically significant results. Mel, melatonin; Ser, serotonin; Dop, dopamine.

* P < 0.1

** P < 0.05

*** P < 0.01

**** P < 0.001

All other ratios were nonsignificant and are not shown.

## Discussion

The results of the present study can be summarised as follows: (i) the 24-h patterns of plasma melatonin concentrations during the four seasons of the year in Cushingoid animals were similar to those in controls; (ii) plasma serotonin profiles were affected by season, and serotonin concentrations tended to be lower in Cushingoid horses in summer (June) and winter (December); (iii) the total amount of dopamine released was dependent on season and markedly lower in Cushingoid versus control horses; (iv) total amounts of cortisol in plasma throughout the 24-h profile were significantly higher in diseased horses only in June; and (v) large changes, mostly reductions, were seen in the serotonin : melatonin and dopamine : melatonin ratios in the diseased animals. Although our investigation does not discriminate between the effects that these circulating plasma neurohormones may have on the peripheral and/or central dopaminergic systems implicated in the pathogenesis of Cushing’s disease, our results do provide evidence for possible direct or indirect contributory effects from dopamine and serotonin.

To the best of our knowledge, this is the first systematic study that has measured plasma concentrations of melatonin, serotonin, dopamine and cortisol simultaneously across a 24-h period at four seasons of the year in horses with Cushing’s disease and age-matched controls. Our results, even for control animals, are difficult to compare with those in the literature because of the paucity of relevant published information.

The 24-h melatonin output was affected by season of year, reflecting the increased duration of the nocturnal peak during the short days of autumn and winter. Nocturnal increases in melatonin, which were longer during longer nights, have been seen previously (19, 20) but no patterns were reported for the light period. Diekman *et al.* (21) compared seasonal variations in the concentration of melatonin in peripheral blood in light and dark, but noted a nocturnal rise only in June. The mean (or median) values for the plasma concentration of melatonin in all the above studies were very similar to those in Fig. 1. Our results in horses with Cushing’s disease showed that their melatonin rhythm matched the light/dark cycle across the year in the same way as it did in controls, which indicates that an inability of the animals to ‘read’ the time of the year is unlikely to be the cause of the disease. This important observation demonstrates that the translation of photoperiodic information is maintained in horses suffering from PPID and, therefore, does not support our original working hypothesis.

In a previous study, peripheral plasma concentrations of serotonin were measured in summer, autumn, winter and spring in clinically normal ponies and those predisposed to laminitis, and no significant differences were observed (22). Although light/dark differences were not investigated in the latter work, nyctohemeral increases in serum serotonin in the healthy, athletic horse have been reported (23). The present study shows that the concentration of free serotonin is decreased at specific times of the year in horses with Cushing’s disease. Although the reasons for this reduction remain unknown, enhanced conversion of serotonin to melatonin could account, at least in part, for the lowered circulating level. It should be noted that the amount of serotonin detected in plasma depends on the number of platelets present (24) and is much lower in platelet-poor samples, such as determined in the present study and the previous study by Bailey *et al.* (22).

Plasma dopamine concentrations were significantly decreased at specific times of the year in horses with Cushing’s disease. In a previous study, an over 80% decline in the content of this neurohormone (as well as of its metabolites) had been observed in the equine pituitary pars intermedia of Cushingoid animals (25). Similarly, McFarlane *et al.* (3, 26) reported that horses with PPID had five-fold less tyrosine hydroxylase staining and increased levels of 3-nitrotyrosine, a marker for oxidative stress. This suggests that a decreased central content of dopamine and reduced dopaminergic tone may play a role in the pathogenesis of Cushing’s disease. Whether or not the reduced level of circulating hormone observed in the present study is a consequence of the decrease in the pituitary pars intermedia cannot be resolved on the basis of the present experiments. Notwithstanding, our observations are consistent with previous findings that administration of dopamine agonists benefits the PPID horse (6, 7).

Equine plasma concentrations of cortisol were similar to those found in previous studies (27–29) and did not demonstrate the circadian rhythm described by some investigators (28). However, Irvine and Alexander (28) showed that the circadian rhythm of cortisol can easily be obliterated in normal horses, but not trained racehorses, by minor perturbations in the environment. Moreover, it is possible that increased age of the animal is also one of the obliterating factors. Nevertheless, it was interesting to find that the concentrations of this hormone were increased in Cushingoid horses only during the summer, which corroborates the selective alteration of the hypothalamic-pituitary-adrenal axis at this time of the year.

It is important to note that, in the present study, three factors may have obscured, or minimised, differences between the Cushingoid and control horses: the relatively small number of experimental animals, a large inter-animal variation (encountered in most studies on large live, whole mammals) and the specific properties of the protocol imposed by the UK Animal Welfare regulations which did not allow blood sampling of individuals every 3 h. To address one of these issues, we evaluated the serotonin : melatonin and dopamine : melatonin ratios using the same statistical methods. It is well known that ratios are less sensitive to inter-subject variations and that a number of physiological processes depend on a ratio of two effectors and not their absolute levels. Analyses of these additional results provide strong support for our arguments.

In conclusion, comparisons of circadian rhythms for melatonin, serotonin, dopamine and cortisol in four seasons of the year in horses with Cushing’s disease and their age-matched controls implicate both dopamine and serotonin in the pathogenesis of the disease. The similar durations of the nocturnal melatonin peaks in both groups of animals suggests that photoperiodic information in Cushingoid horses can be processed normally and, therefore, that impaired melatonin output is unlikely to be the reason for the circannual manifestation of the disease.
